# Genetics of Marbling in Wagyu Revealed by the Melting Temperature of Intramuscular and Subcutaneous Lipids

**DOI:** 10.1155/2017/3948408

**Published:** 2017-10-23

**Authors:** Sally S. Lloyd, Jose L. Valenzuela, Edward J. Steele, Roger L. Dawkins

**Affiliations:** ^1^CY O'Connor ERADE Village Foundation, P.O. Box 5100, Canning Vale South, WA 6155, Australia; ^2^CY O'Connor Centre for Innovation in Agriculture, Murdoch University, 5 Del Park Road, Box 1, North Dandalup, WA 6207, Australia; ^3^Melaleuka Stud, 24 Genomics Rise, Piara Waters, WA 6112, Australia

## Abstract

Extreme marbling or intramuscular deposition of lipid is associated with Wagyu breeds and is therefore assumed to be largely inherited. However, even within 100% full blood Wagyu prepared under standard conditions, there is unpredictable scatter of the degree of marbling. Here, we evaluate melting temperature (*T*_*m*_) of intramuscular fat as an alternative to visual scores of marbling. We show that “long fed” Wagyu generally has *T*_*m*_ below body temperature but with a considerable range under standardized conditions. Individual sires have a major impact indicating that the variation is genetic rather than environmental or random error. In order to measure differences of lower marbling breeds and at shorter feeding periods, we have compared *T*_*m*_ in subcutaneous fat samples from over the striploin. Supplementary feeding for 100 to 150 days leads to a rapid decrease in *T*_*m*_ of 50% Red Wagyu (Akaushi) : 50% European crosses, when compared to 100% European. This improvement indicates that the genetic effect of Wagyu is useful, predictable, and highly penetrant. Contemporaneous DNA extraction does not affect the measurement of *T*_*m*_. Thus, provenance can be traced and substitution can be eliminated in a simple and cost-effective manner.

## 1. Introduction

Marbling (or the accumulation of intramuscular fat) is the holy grail for beef producers, chefs, and their customers, but there is still no agreed definition and therefore no universal standard of measurement [1, 2]. So as to increase commercial returns based on superior taste and health benefits, there have been countless attempts to improve the reproducibility of visual and scanning scores but with limited success [1, 3].

Lipid profiles of highly marbled samples have revealed a high content of oleic acid and therefore a reduction in melting temperature (*T*_*m*_) [4–6]. A precise and high throughput method for the measurement of *T*_*m*_ exists [7] and is used here to interrogate the complex interplay between the genetic and environmental factors which can be optimized by the producer to the benefit of the health conscious consumer.

Because of the association of Wagyu breeds with high marbling and high oleic acid content, these traits can be assumed to be genetically determined and faithfully inherited [8, 9]. However, in spite of numerous studies [10–19], it has not been possible, hitherto, to identify markers which allow a breeder to quantify superior genetics in individual sires and dams. Some of the explanations for the slow progress include the following:

- Complexity due to interactions of several metabolic processes and their regulatory mechanisms [6, 20, 21].
- Contribution of many genes with small effects [22].
- Uncontrolled environment factors associated with supplementary feeding [23–25].
- Difficulty in quantifying marbling reproducibly [2].
- Unreliable tracing of meat from paddock to plate.
- Perception that fat is dangerous.

Recently it has been demonstrated that that low fat diets have not improved health [26]. In fact, higher oleic acid and therefore low *T*_*m*_ are preferable in terms of lipid profiles [27–29]. This has led to the increasing popularity of the Wagyu brand worldwide. Not surprisingly, mislabeling is now rife resulting in the need to be able to confirm the provenance of retail samples.

Here we show that low *T*_*m*_ is heritable and that the same fat sample can be used for the DNA tracing without affecting the measurement of *T*_*m*_.

## 2. Materials and Methods

Postmortem samples of meat and fat were taken from carcasses of animals harvested for routine food production. Therefore, ethics approval was not required.

### 2.1. Dataset 1 Full Blood Wagyu with Identified Sires

Two cohorts of Wagyu steers (*n* = 126) were fed for 300 ± 20 days with a proprietary ration within the same commercial feedlot. One-gram samples of meat from the longissimus dorsi were taken from between the 10th and 11th rib. AUS-MEAT marbling score (MS) was scored between the 10th and 11th rib, with an average of 7.5 and a range from 2 to 11. Steers for the comparison of sires had their paternity confirmed by DNA testing [30]. Only one progeny of each dam was included so as to focus on the effect of the sire.

### 2.2. Dataset 2 European and Wagyu Cross Breeds with Varied Feed Time

Melaleuka Stud, located in the Peel region of Western Australian, 100 km south of Perth, runs a variety of European breeds including Simmental, Gelbvieh, and Angus. This herd was selected to produce high quality beef on pasture, finished with 2 to 4 months of supplemental feeding. Black and Red Wagyu (full blood or pure bred) have been mated with these European breeds.

Calves stay on milk until 4 months of age when they are weaned and male calves are castrated. After weaning, they continue grazing Kikuyu and Ryegrass pasture until they reach 300 kg. Their feed is then supplemented with 9 mm EasyBeef pellets (Milne Feeds, Perth, Australia)* ad libitum*. The main ingredients of the EasyBeef pellets are lupins, barley, oats, wheat, and triticale. The nutritional composition, based on dry matter, is crude protein (min) 14.5%, metabolizable energy (est.) 11.0 MJ/kg., crude fiber (max) 20.0%, urea (max) 1.5%, and monensin 26.6 ppm.

The feeders are considered ready for slaughter when they reach a weight of 400 kg and are slaughtered to match demand. Some animals were kept on feed longer to test the effect of increased feeding on *T*_*m*_ and meat quality. The average live weight at slaughter for animals in this study was 461 kg, average age at slaughter was 15.4 months (range 8 to 23), and the average days on feed was 104 days (range 17 to 288). Body numbers from abattoirs were matched to farm records and pedigrees via their RFID tags, where possible identity was confirmed by in-house proprietary DNA testing [30].

Subcutaneous fat overlying the striploin (HAM number 2140) of these cattle was collected after boning and wet aging for 1 to 3 weeks.

### 2.3. Fat Extraction and *T*_*m*_ Measurement

Intramuscular fat was extracted from dataset 1 samples by digestion with proteinase K. This method allows for simultaneous extraction of intramuscular fat and DNA from 0.5 gram samples of meat if the fat content is above 20%. The samples were incubated at 56°C, digested in a proteinase K mixture for 4 hours, and centrifuged at 10,000 ×g for 2 minutes to separate the fat from the dissolved DNA and protein solution. Fat was removed for *T*_*m*_ measurement by pipette. DNA was extracted from the remaining mixture using a standard salting out method.

Intramuscular fat content for many of the carcasses of dataset 2 was too low to allow extraction by the above method. Instead, fat was extracted from 1-gram samples of subcutaneous fat by rendering at 90°C for at least eight hours.

Samples from 17 sirloin steaks with intramuscular fat higher than 20% were used to determine whether fat separated during a DNA extraction process could be used for *T*_*m*_ measurement. Fat was extracted by both digestion and rendering from the same samples and the *T*_*m*_ measurements compared.

*T*_*m*_ of all fat samples was determined in triplicate according to the thermocycler method [7], which is closely correlated to slip points, although the values are higher by 2°C for animal fat with a *T*_*m*_ of 40°C.

## 3. Results

### 3.1. *T*_*m*_ Is Affected by Sire

**Figure 1:**
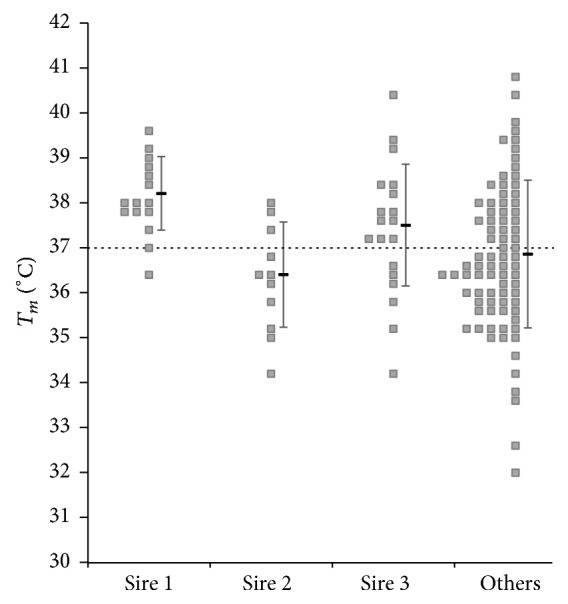
*T*_*m*_ distributions of Wagyu carcasses differ by sire. The melting temperature of intramuscular fat samples taken from between the 10th and 11th rib of 126 carcasses of full blood Wagyu steers. All animals were fed the same ration for 300 ± 20 days. Individual *T*_*m*_ measurements of carcasses are grouped by sire (mean and standard deviation). Animals with either an uncertain sire or a sire with less than 10 progeny are grouped under “other” sires. Progeny of Sire 3 shows considerable scatter, whereas 8/11 of those of Sire 2 are below 37 degrees compared with 1/15 in the case of Sire 1. The difference between Sire 1 and Sire 2 is statistically significant with a chi-square statistic of 12.2 and thus a *p* value < 0.01.

Samples were taken from long fed Wagyu steers differing only by sire and dam (dataset 1). The steers were fed, harvested, and tested in two cohorts two months apart. The cohorts did not differ significantly in feeding, genetics, or initial *T*_*m*_ (as shown in Supplemental Table  1 in Supplementary Material available online at https://doi.org/10.1155/2017/3948408) and have therefore been combined for further analysis. *T*_*m*_ and marble score were analyzed by sire for the three sires with more than ten progenies. As shown in Figure 1, *T*_*m*_ of the progeny of Sire 2 fell consistently, whereas Sire 1 had little impact. In fact, 14 progenies of Sire 1 were above 37°C, compared to only 3 of Sire 2. The cross-product ratio is 104/6 or 17, as shown in Figure 1. This difference is highly significant (*p* value < 0.01 by *χ*^2^). It is noteworthy that there is more scatter with Sire 3 and all remaining sires.

By contrast with *T*_*m*_, visual scores of marbling gave greater scatter, did not demonstrate a sire effect, and must be misleading in their present form.

### 3.2. In Wagyu, *T*_*m*_ Falls with Days on Feed and Proportion of Wagyu

**Figure 2:**
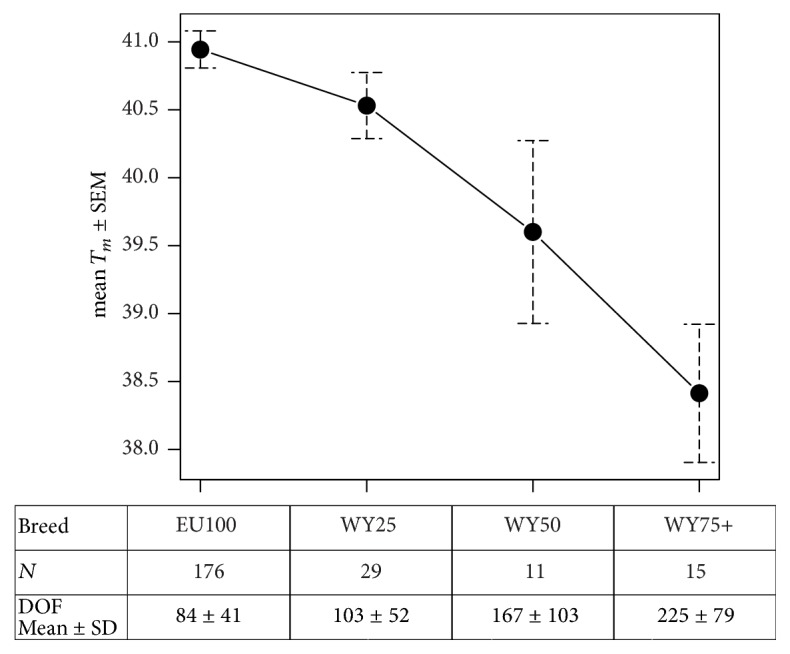
*T*_*m*_ *decreases with feeding and increasing proportion of Wagyu ancestry*. *T*_*m*_ of subcutaneous fat samples over the loin of a mix of breeds and crossbreeds including Simmental, Gelbvieh, Angus, Dexter, and Wagyu. 176 samples (EU100) came from 100% European breeds fed for an average of 81 days. WY25, WY50, and WY75+ samples had 25%, 50%, and 75–100% Wagyu ancestry, respectively. There were 29 samples of WY25, 14 samples of WY50, and 11 samples of WY75+ with average days on feed of 103, 167, and 225, respectively.

Notwithstanding the genetic effects, there is also a major environmental effect on *T*_*m*_ and marbling. *T*_*m*_ results of dataset 2, grouped by proportion of black Wagyu, are shown in Figure 2. *T*_*m*_ falls with increase in Wagyu and days on feed. Separating these two variables is not yet possible but, in the meanwhile, the results suggest that increasing the content of Wagyu genes allows the benefit of long feeding. The European cattle included in this study do not show the same benefit as the Wagyu.

Importantly, the benefits are seen with only 25% Wagyu, again emphasizing the high penetrance of the Wagyu genetics.

### 3.3. Quantitative Effect of Feeding

**Figure 3:**
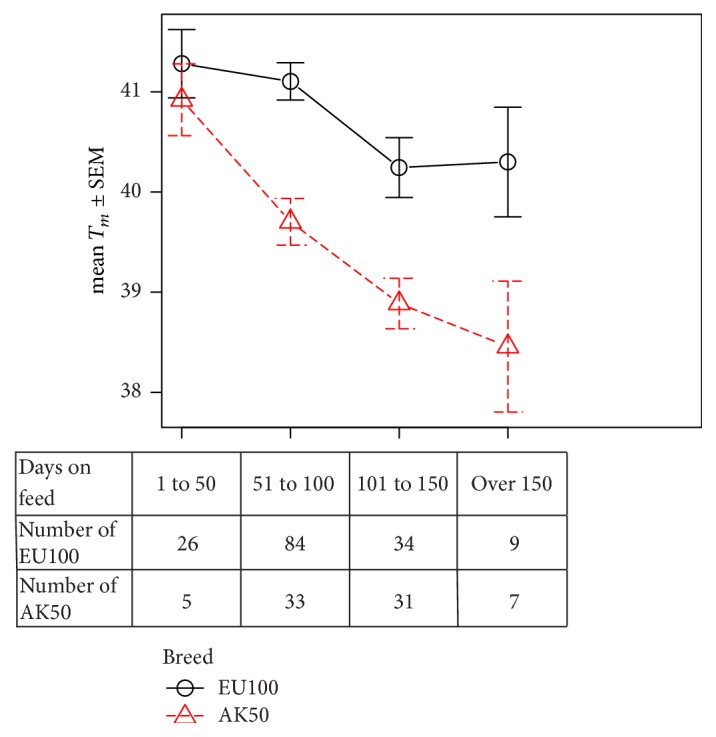
Red Wagyu sired carcasses have lower *T*_*m*_ for equivalent DOF. *T*_*m*_ was measured for subcutaneous fat samples taken from the loins of 229 carcasses. The cattle were backgrounded on pasture and then fed on pellets until they reached a satisfactory weight and fatness. The results are grouped by days on feed and by breed of sire (European or Akaushi). The dams of all carcasses were European breeds. Breed and days on feed were both statistically significant influences on *T*_*m*_, with *p* < 0.01 calculated by multiway ANOVA. The difference between the two groups was significant after only 51–100 days on feed.

So as to address the complex interaction between genetics and environment, we compared two breed groups from dataset 2: a control group of purely European cattle (EU100) and the F1 Red Wagyu, also known as Akaushi and recorded as AK50. The dams have a similar breed composition and history to the EU100 control group. So as to avoid the complexity of sampling intramuscular fat before it is visible, we have relied on *T*_*m*_ measurements of overlying subcutaneous fat. The effect of feeding is clear as shown in Figure 3. *T*_*m*_ falls progressively even with only a 50% infusion of Akaushi.

### 3.4. DNA Extraction Does Not Invalidate Measurement of *T*_*m*_

**Figure 4:**
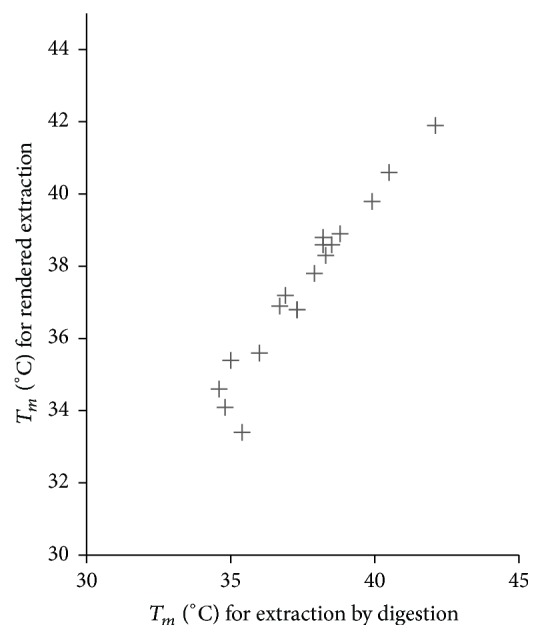
Simultaneous extraction of fat and DNA does not change *T*_*m*_. There is excellent correlation between *T*_*m*_ measurements of fat harvested during DNA and extracted by rendering (Pearson's *R* = 0.97). There was no measurable bias (mean difference 0.13, SEM = 0.14). Either extraction method can be used for direct comparison without adjustment.

In Figure 4 we show that extracting DNA with the proteinase K does not affect the measurement of *T*_*m*_ on the same extract. Oxidation of the polyunsaturated fatty acids in the sample that may have occurred during rendering at 90°C did not have a measurable effect on the melting point, as expected [4, 31].

## 4. Discussion

The intention of these studies is to resolve, in part, the manifest confusion facing producers and consumers of healthy beef.

It is clear that Wagyu beef is superior, as reflected by the commercial returns for highly marbled beef, but increasingly the brand is amenable to misuse.

A major issue is the lack of a reproducible measurement of the degree of marbling. Multiple and incompatible systems of scoring may have been retained perhaps to the advantage of some sectors. The measurement of *T*_*m*_ is possible at successive stages of the production line so that quality can be confirmed. At the same time, DNA can be extracted so that provenance can be confirmed.

The difficulty faced by the breeder is even more important. Nonreproducible measurement obfuscates attempt to identify breeding values and therefore confound selection of superior sires. This issue becomes particularly important in an attempt to upgrade first crosses.

The present results show that even WY25 can have reduced *T*_*m*_ but the scatter is substantial leading to lack of consistency. Future studies may identify those sires which are well suited to crossbreeding.

So as to reduce the number of variables we sampled AK50 at differing days on feed. All had European dams. The initial results are promising in that there was a progressive decline in *T*_*m*_. Further work may define the preferred type and length of supplementary feeding. Importantly, there is also the potential to examine the controversies surrounding the use of grass versus grain. Whilst there is growing consumer demand for less intensive feeding and especially for “grass-fed,” there is also the perception that corn and perhaps other grains are necessary for extreme marbling. Given reproducible measurements, it should be possible to define acceptable compromises between supplementation, on the one hand, and tastiness and healthiness, on the other hand.

A major finding of this study is the difference in *T*_*m*_ between the progeny of two full blood Wagyu sires. Sire 1 and Sire 2 share a paternal and a maternal grand sire and were imported from the same prefecture in Japan. Pedigree analysis alone would not predict large differences in lipid composition. It is noteworthy however that Sire 1 and Sire 2 are quite different in their C19 haplotypes, as described elsewhere [32]. A major issue remains unresolved. The degree of marbling and the lipid profile differ depending upon the site of sampling; as an approximation the intramuscular accumulation of lipid progress from the brisket backwards with the more caudal fat deposits having somewhat lower proportions of oleic acid and higher *T*_*m*_ [33]. Therefore, comparable samples need to be from a fixed location. Even within the same muscle group there is variation depending upon sampling [34]. We recommend further experience using subcutaneous fat so that its utility can be extended. Ultimately, it should be possible to take in vivo samples so as to monitor changes with time, genetics, and feed.

## Supplementary Material

Supplemental Table 1 Comparison of the two cohorts of data set 1 show no significant differences in *T*_*m*_ or marble scores. The three sires each have progeny in both cohorts.
