# Supplementary material for: Genetics of Marbling in Wagyu Revealed by the Melting Temperature of Intramuscular and Subcutaneous Lipids
Source: Int J Food Sci. 2017 Oct 23;2017:3948408. doi: 10.1155/2017/3948408 (PMC5672612; doi:10.1155/2017/3948408)
Supplement: Supplementary file 1 — Supplemental Table 1 Comparison of the two cohorts of data set 1 show no significant differences in Tm or marble scores. The three sires each have progeny in both cohorts. [file 3948408.f1.pdf]

Tables

Supplemental Table S1: Cohort summary

	Tm		MS		No. Sire 1	No. Sire 2	No. Sire 3	Total N
	Average	Range	Average	Range				
**Cohort 1**	37.1	32.1-41.0	7.1	2-11	10	8	8	64
**Cohort 2**	37.1	32.7-40.5	7.9	5-11	5	3	11	62
**Total**	37.1	32.1-41.0	7.5	2-11	15	11	19	126
